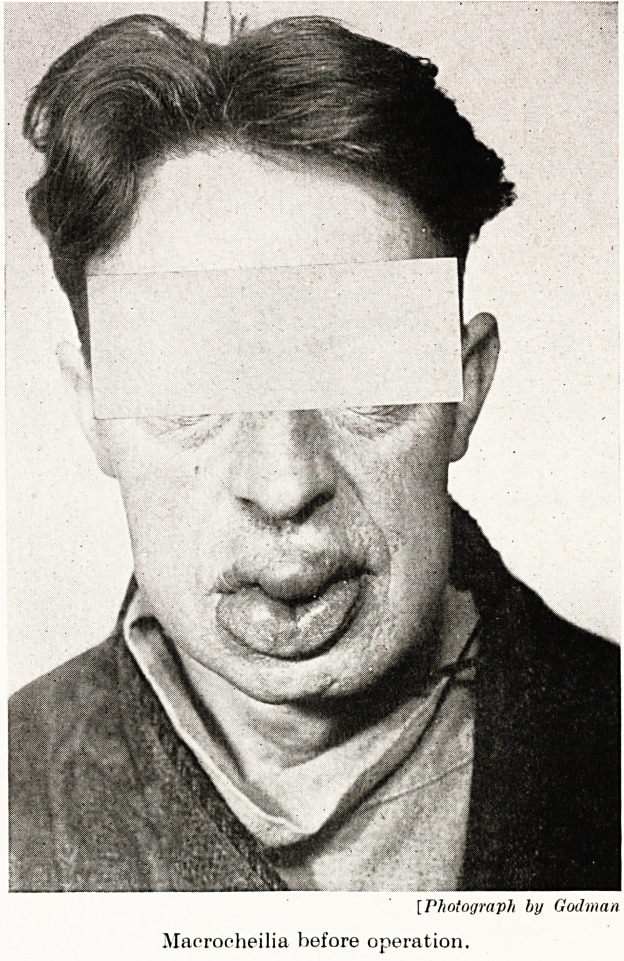# A Case of Macrocheilia

**Published:** 1948

**Authors:** D. C. Bodenham


PLATE V

[Photograph by Godman

Macrocheilia before operation.

### A CASE OF MACROCHEILIA

BY

D. C. Bodenham, M.B., F.R.C.S.

Mr. B. age 42 years. Eight years ago the patient underwent an operation for extensive removal of bilateral tuberculous glands of neck. This was followed by temporary oedema of the face and lips, and led to a lip sucking habit which persisted until recently.

In August, 1947, the patient was admitted to a medical ward for treatment of a gastric ulcer. At this time the continued sucking had led to enormous enlargement of the lips and not till then did he ask if anything could be done to improve his condition. The lips showed enormous hypertrophy, particularly of that part which lay within the mouth. Lymphatic drainage through the neck now appeared to be adequate. There was no facial oedema.

*Operation.* A wedge of mucosa and hypertrophied submucosal tissue was removed by intra-oral incision extending horizontally across the whole width of the lower lip. After the correct volume of tissue had been excised, the defect was closed. The same procedure was carried out in the upper lip. This reduction in size removed the stimulus to lip sucking and six months later the result is very satisfactory. I am grateful to Dr. R. C. Clarke for referring the case for treatment and to Mr. G. M. FitzGibbon for allowing me to treat the case.

## Figures and Tables

**Figure f1:**